# Letter to the Editor

**DOI:** 10.1177/11786329231188045

**Published:** 2023-07-17

**Authors:** François Maneraguha Kajiramugabi

**Affiliations:** Faculty of Nursing, University of Montreal, Montreal, QC, Canada

Dear Editor,

We read with great interest Tilahun et al’s paper^1^ published in your journal in 2022.

We would like to highlight the authors’ research relevance on a timely topic, access by youth to education and reliable information about sexual and reproductive health services. Globally for instance, the proportion of young people who access comprehensive information on sexual health issues is low, 34%, despite their right to comprehensive sex education.^2^ This is a reason to investigate this topic further. However, we wish to point out some methodological issues that could undermine the rigor of mixed methods health research and lead to inappropriate conclusions, often bringing healthcare quality into disrepute.

Indeed, Tilahun et al^1^ claim that they conducted a mixed methods cross-sectional study including a community-based cross-sectional and qualitative study. But the title only indicates that it was a community-based cross-sectional study. In this regard, it’s strongly suggested by various authors that the words *mixed methods* be included in the title to let readers know in advance what method was used. Thus, Creswell and Plano Clark^3^ (p. 146) assert: « *a good mixed methods title includes the word mixed methods to highlight the overall approach being used* ».

In a journal paper, the need to use a mixed method and specify the study design should be incorporated and justified in the problem statement.^3^ This does not appear in Tilahun et al’s paper.^1^ Furthermore, and contrary to other researchers’ suggestions,^3-5^ Tilahun et al^1^ did not specify whether the design was convergent, explanatory sequential, exploratory sequential, or complex that is, mixed methods participatory and social justice, experimental, evaluation, or case studies. This lack of precision, explicit, and persuasive rationale for methodological approach choice compromises data interpretation and inferences.^4,5^ Yet, mixed methods research advantages^3^ are now well recognized, including its ability to provide comprehensive answers to complex research problems^4^ such as youth’s equitable access to high-quality sexual health services and information.

Fortin and Gagnon^4^ consider any methodological combination to be mixed methods research when 3 essential characteristics are met: at least one qualitative and one quantitative method are combined, each method is rigorously used, and data collection, analysis, and/or results are integrated. Integration can be done either by connecting phases, comparing results, or assimilating data through 9 strategies.^4^ This perspective is shared by Polit and Beck.^5^ Creswell and Plano Clark^3^ also recommend that researchers achieve integration at analysis and interpretation stage using mixing, merging, and connecting as strategies. Creamer^6^ goes further by advocating full integration in planning and design, data collection, sampling, analysis, and inference development. Therefore, it appears that Tilahun et al^1^ haven’t specified in their study qualitative and quantitative data integration in any of above stages as recommended.^3-6^ The authors’ 3 explanatory sentences for the study qualitative component^1^ (p. 2) do not imply integration:

“*A qualitative study was included to support the findings of the quantitative study. A qualitative study was also meant to address the perspectives of providers. A qualitative study was conducted using focus group discussions (FGDs), in-depth interviews (IDs), and observations of health facilities that provide youth sexual and reproductive health services.*”

Even less so, authors^1^ did not precise how qualitative interviews explained quantitative results of their study. Nor did they indicate whether qualitative and quantitative results converged or diverged.^3^ This simple juxtaposition of quantitative and qualitative methods without integration of data collection, analysis, or results raises questions about Tilahun et al’s study^1^ given that integration is the mixed methods research “backbone” or central feature.^5^ In support of this view, Creswell and Plano Clark^3^ state (p. 18): “*Most important in this list is the utilization of two sets of data, one quantitative and one qualitative, and the integration of these data*.”

Ultimately, we hope these comments will be helpful in thinking deeply about mixed methods research strengths and challenges in health and nursing, especially with youth seeking reliable information and appropriate sexual health services. It’s essential for researchers wishing to use mixed methods to provide a clear rationale for their choice, write a specific title indicating the use of mixed methods research, specify the research design to be used, and prioritize the integration of qualitative and quantitative data. This would help to promote the rigor, value, and effectiveness of mixed methods research to generate higher quality knowledge to inform health and nursing policy and practice.
